# Chewing side preference, facial asymmetry and related factors in the Northern Finland birth cohort 1986

**DOI:** 10.2340/aos.v83.41392

**Published:** 2024-09-18

**Authors:** Elina V. Heikkinen, Ville Vuollo, Tuomo Heikkinen, Virpi Harila

**Affiliations:** a Research Unit of Population Health, Faculty of Medicine, University of Oulu, Oulu, Finland; b Medical Research Center Oulu, Oulu University Hospital, Oulu, Finland

**Keywords:** 3D imaging, cohort study, facial asymmetry, preferred chewing side

## Abstract

**Objective:**

The aim of this study was to find out how the preferred chewing side (PCS) affects facial asymmetry, what kind of factors affect PCS and whether there are differences in facial asymmetry between symmetrical and asymmetrical masticators.

**Material and methods:**

The study included 748 subjects (females *n* = 452, males *n* = 296) born in 1985–1986 in Northern Finland (Northern Finland Birth Cohort 1986, NFBC 1986). Subjects’ faces were captured in facial 3D images with stereophotogrammetry technology, and they filled in a questionnaire concerning oral health. A comprehensive dental examination was done by a dentist. Subject’s chewing side preference was studied by chewing a piece of paraffin, cotton roll or parafilm. Asymmetry was measured from 3D images with different asymmetry measurements and facial landmarks.

**Results:**

Reduced number of teeth on contralateral side affects PCS (odds ratio [OR] = 2.44 in the case of one tooth is missing). Being female increased the whole face and lower face symmetry (*p* < 0.001–0.824). Self-reported temporomandibular disorders (TMD) pain has an effect on the sidedness of the chin; there is more pain in the larger side of the chin (OR = 9.45). Different chewing materials had no significant effect on the proportion of chewing sides.

**Conclusions:**

Females have a more symmetrical face compared to males. PCS does not have a statistically significant effect on facial asymmetry, but the variable affecting PCS itself is extracted teeth.

## Introduction

Asymmetry in the face has always been an interest among orthodontists. Previous studies have found that the preferred chewing side (PCS) affects facial asymmetry, but with contradictory conclusions. Some researchers suggest that PCS and a larger chin side locate on opposite sides in the face, whereas others propose that PCS and a larger hemiface correlate [1, 2]. On the other hand, both studies have a cross-sectional design so the effect of time cannot be considered.

The limit value to separate asymmetry from symmetry is two mm in lateral deviation. Four mm is thought to be a border to differentiate asymmetry and symmetry measured from the tip of the chin, the Menton landmark [3, 4]. One indication for surgical intervention is severe and progressive facial asymmetry. Jaw surgery is indicated if there is more than five degrees’ deviation between the transversal occlusal plane of the maxilla and the plane of the base of the eyes. However, jaw surgery, usually including both the maxilla and mandible, is only a secondary form of treatment; other, more non-invasive treatments are the primary treatment [5].

During adolescence, facial growth is symmetric among healthy individuals [6]. In the course of time, the amount of facial asymmetry has been found to increase [7]. Contrary to that, more facial asymmetry has been found in infants compared to children and adults in some studies [8, 9]. Facial asymmetry as a laterality, like handedness and footedness, may have a genetic background. The right-sided dominance and left-sided weakness of the jaw are quite a common finding, and it is independent of age, sex and skeletal relationships [3, 10–13]. The origin of laterality derives from living organisms’ fundamental asymmetry and molecular biology [14]. Environmental factors have also been observed to have an effect on facial asymmetry. Environmental factors have the biggest effect on horizontal facial asymmetry and mandibular ramus height while heritable traits concentrate in the area of the nose and lips [15]. The lower two thirds of the face have been found to be more asymmetric compared to the upper face [3, 16–18], but there are studies suggesting that all parts of the face have the same amount of three-dimensional symmetry [19]. The aetiology of facial asymmetry is thus complicated, and none of the factors is more dominant than the others.

However, one possible aetiological factor is PCS [20]. Most people chew more on one particular side, that is, the right side [21–23]. Different variables, such as dental wear, affect PCS, but no connection has been found between caries or missing teeth and PCS [24–26]. Occlusal parameters affect PCS, but there is disagreement about the effect of crossbite on PCS [21, 27–29].

To our knowledge, we are the first to study this topic with large cohort material. There is a lack of information on how PCS affects facial asymmetry in larger samples. Clinicians can utilise information on the effect of PCS on facial asymmetry as an aetiological factor when making orthodontic treatment plans or considering the consistency of a received outcome. In basic dental care, the results of this study serve as base information, which can be exploited when considering the treatment of temporomandibular disorders, for example [1, 3, 20]. The aim of this cohort study is to find out how PCS affects facial asymmetry, what kind of factors affect PCS and whether there are differences in facial asymmetry between symmetrical and asymmetrical chewers by means of facial 3D images.

## Materials and methods

The study population consist of 9,362 mothers, whose children (*n* = 9,479) were born in 1985–1986 in Northern Finland (Northern Finland Birth Cohort 1986, NFBC 1986) [30]. The cohort includes all the children whose expected date of birth was between July 1, 1985 and June 30, 1986 in Oulu and Lapland, the two northernmost provinces in Finland. A small percentage of births took place at the end of June 1985 and at the beginning of July 1986. The cohort population has been followed during their whole life, beginning from pregnancy and prenatal time and continuing to 33–35-year follow-up, where there was total number of 1,807 participants [31]. *In situ* at cohort collection, PCS examination was done to 748 participants. The age of the subjects was 32.9–35.5 years, and the mean age was 34.2 years. The proportion of females and males was 60.4 and 39.6%, respectively.

In the 33–35-year follow-up (5/2019–12/2020), subjects’ faces were captured in facial 3D images with stereophotogrammetry technology, and they filled in a questionnaire concerning oral health. The faces of the subjects were recorded by a 3dMDFace system (3dMD, Atlanta). They were asked to look straight ahead and assume a neutral facial expression. The facial position was standardised by setting the subjects in the same reference frame, and the origin was set as a point halfway between the endocanthion of the left and right eye [32]. Pose standardisation and image procession were done with Rapidform2006 software (INUS Technology, Inc., Seoul, South Korea). Subjects with long facial hair were excluded because of rough surface on the chin area.

**Figure 1:**
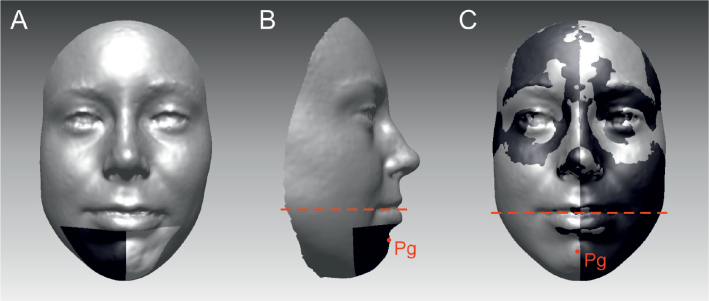
(A) Regions for calculating CVAS and areas used calculating sidedness of the chin. (B) The Pogonion landmark and the area of the lower jaw, which have been used for calculating AD and SP. CVAS division viewed from the side. (C) The Pogonion landmark and the area of the lower jaw. The original 3D model and the mirrored model as superimposed.

Next two asymmetry scores were measured by the widely used method as in the study of Djordjevic et al. [6]. Each original 3D face was mirrored across the sagittal plane (YZ plane). The surfaces above the subnasale landmark of the original model and the mirrored one were then superimposed with the iterative closest point algorithm. The average distance (AD) between the original and mirrored 3D model was calculated for the whole face and separately for the lower face (below the mid-lip line). Furthermore, the matching of the original and mirrored face was measured by the symmetry percentage (SP) of the facial area where the distance from the mirrored surface did not exceed 0.5 mm. SP was also analysed separately for the whole face and lower face. The greater the AD value, the more asymmetry the face has, whereas the greater SP value means a more symmetrical face. Chin volume asymmetry score (CVAS) is obtained by dividing volume of the larger chin side by the smaller ones, like our earlier study defines it (2) (Figure 1). The absolute value of the x coordinate of the pogonion (Pg) point on the chin was also used for measuring lower face asymmetry (Figure 1). Also, other facial landmarks were checked from lip area (labiale inferius, labiale superius, mid cheilion, mid christa philtri).

At first, PCS was studied by asking the subject directly, first orally from 57 subjects; after that, the question was part of the oral health questionnaire. The question was: Which side in the mouth do you prefer when chewing food? The alternatives were 1. Right, 2. Both sides, 3. Left and 4. I cannot say yet (it becomes clear at the next step). It was possible to choose only one alternative. If the subject could not give an answer, the examiner asked them to consider it during stimulated saliva sample collection (part of cohort tests), where the subject chewed on a piece of paraffin (1 g, Orion Diagnostica, Espoo, Finland), 1/4 of a cotton roll or a 5 cm × 5 cm piece of parafilm (Parafilm ‘M’ Laboratory Film, Bemis) for 5 min. of the subjects chewed paraffin, 236 chewed cotton roll and 362 chewed parafilm. After that, PCS was enquired again, and if the subject could not answer, it was recorded that the subject used both sides for chewing. A total of 748 answers were received. Different chewing materials had no significant effect on the frequency distribution of chewing sides.

Five trained and calibrated Finnish dentists performed standardised clinical dental examinations in this large cohort study 5/2019–12/2020. The examination included the whole dental status as well as a clinical stomatognathic examination. Caries and cracks, fillings and extracted teeth were looked at separately. Caries and fillings were checked for five surfaces of each tooth. Caries was classified according to the International Caries Detection and Assessment System (ICDAS, categories 0–6). Wear includes attrition and erosion categorised as BEWE 3 [33]. Attrition was checked tooth specifically and erosion as dental sextants. For extracted teeth, the wisdom teeth have been excluded because those are generally quite often extracted teeth. Molars, premolars and canines as extracted teeth were considered equal in the analyses. Missing teeth were counted if they had not been replaced by a prosthetic structure. In temporomandibular disorders (TMD) pain and headache, self-reported pain and TMD diagnoses were looked at separately according to the Diagnostic Criteria for Temporomandibular Disorders (DC/TMD) [34]. Self-reported TMD pain and headache was asked during the last 30 days, but unfortunately, the duration and severity information of the pain are not included in the analyses. There are seven self-reported pains: m. temporalis, m. masseter, other masticatory muscle, temporomandibular joint, no masticatory muscle or TMJ, temporal headache and other headaches. The number of TMD diagnoses is also seven. The diagnoses were disc displacement with reduction, disc displacement without reduction with limited opening, degenerative joint disease, myalgia, myofascial pain with referral, arthralgia and headache attributed to TMD. To examine the sidedness of variables affecting PCS, a right minus left side subtraction was done for the following variables: caries and cracks, fillings and extracted teeth, sum of TMD diagnoses, wear and self-reported TMD pain. In the analysis, incisors have been excluded for fillings, caries, wear and extracted teeth, because those do not affect PCS presumably as much as premolars and molars do. Also, for caries and cracks only the occlusal surface (surface 1) has been included for final analyses, when estimating variables affecting PCS because predictably it has bigger effect compared to four other tooth surfaces.

### Statistical analysis

Statistical analyses were done using IBM SPSS Statistics software version 26.0 (IBM Corporation, Armonk, New York). Crosstabulations and chi-squared tests were used to examine the prevalence of PCS in NFBC 1986. Multinomial logistic regression was used to estimate the effect of different variables affecting PCS. The effect of symmetrical chewing on facial asymmetry was examined with linear regression. Logistic regression was used to estimate variables affecting sidedness of the chin. Different combinations of independent variables were experimented with for finding the most suitable model for each dependent variable. Sex was chosen as confounding variable in all regression models. A significant level for *p*-value was chosen to be 0.05.

## Results

Table 1 shows the proportion of PCS between the sexes. Altogether, the right side is used more when chewing compared to the left side, but the most popular answer is chewing on both sides. The differences between males and females are very small.

**Table 1:** Prevalence of PCS in Northern Finland Birth Cohort (NFBC) 1986 study.

Sex	PCS				*p*
	Right *n* (%)	Both sides *n* (%)	Left *n* (%)	Total *n*	
Male	118 (39.9)	136 (45.9)	42 (14.2)	296	0.529
Female	199 (44.0)	194 (42.9)	59 (13.1)	452	
Total	317 (42.4)	330 (44.1)	101 (13.5)	748	

PCS: preferred chewing side.

The prevalence of TMD diagnoses with this material is shown in Supplementary Table 1 (larger, more comprehensive study of the whole NFBC 1986 cohort is going to be published soon). The most frequent diagnosis is unilateral myalgia (6.4%).

One hundred seventy-five of 748 subjects (23.4%) had missing teeth, and of those, 92 subjects (12.3%) had missing teeth on only one side. One hundred twelve subjects had more missing teeth on the other side. There were no differences between genders. It seems that extracted teeth have an effect on PCS (Table 2). Missing one tooth more on the one side makes subjects chew on the opposite side, so more intact side is used when chewing (odds ratio [OR] = 2.44). There was also an impact, when both side chewing was regressed against the left side chewing in the multinomial logit model (Supplementary Table 3). If one tooth more is missing on the left side compared to the right side, OR is 2.44 times more to use both sides while chewing.

**Table 2:** Effect of different variables on PCS using multinomial logistic regression.

PCS	Coefficient	Odds ratio	CI 95 %	*p*
**Right vs. left side**				
Intercept	−0.95	0.39	0.17–0.87	**0.021**
Fillings	−0.10	0.90	0.77–1.05	0.194
Caries	0.15	1.16	0.86–1.57	0.330
Wear	0.17	1.19	0.84–1.68	0.325
Extracted teeth	0.89	2.44	1.35–4.40	**0.003**
TMD diagnoses	−0.30	0.74	0.46–1.19	0.214
Sex	−0.18	0.83	0.51–1.35	0.453

PCS: preferred chewing side, CI: confidence interval, TMD: temporomandibular disorders

Caries: includes also tooth cracks.

Wear: attrition and erosion.

Odds ratio: > 1 increases the odds of having PCS on the opposite side of the variable.

Table 3 describes statistics of asymmetry measurements. Models in Table 4 show that being female makes the whole face, as well as separately, the lower face more symmetric compared to males; AD distances are smaller in females while SP measures are greater than in males. Females have 0.20 mm smaller Pg distance from facial midline compared to males. There is statistically significant difference between females and males in the case of lip area landmarks labiale superius (*p* = 0.020) and mid cheilion (*p* = 0.039). Statistically significant results were not found on the effect of symmetrical chewing on facial asymmetry.

**Table 3:** Descriptive statistics of asymmetry measurements.

	Mean	Std. Deviation	Minimum	Maximum	Median	Lower quartile	Upper quartile
**AD whole face (mm)**							
Total	0.65	0.21	0.30	1.78	0.61	0.50	0.75
Male	0.74	0.23	0.38	1.78	0.69	0.58	0.85
Female	0.62	0.19	0.30	1.54	0.57	0.48	0.71
**AD lower face (mm)**							
Total	1.03	0.59	0.25	3.98	0.87	0.61	1.25
Male	1.16	0.68	0.36	3.98	0.96	0.68	1.46
Female	0.98	0.54	0.25	3.97	0.84	0.60	1.19
**SP whole face (%)**							
Total	54.87	9.73	23.94	81.72	54.86	47.70	61.50
Male	49.77	8.63	23.94	72.45	49.31	43.80	55.94
Female	57.06	9.36	31.61	81.72	57.50	50.30	63.74
**SP lower face (%)**							
Total	37.74	17.86	1.47	86.04	36.71	22.89	49.96
Male	34.47	17.18	1.47	73.84	33.92	19.15	46.01
Female	39.15	17.98	5.74	86.04	38.66	24.98	51.30
**CVAS**							
Total	1.09	0.08	1.00	1.60	1.07	1.03	1.14
Male	1.10	0.08	1.00	1.51	1.07	1.04	1.14
Female	1.09	0.08	1.00	1.60	1.08	1.03	1.14
**Pg distance from midline (mm)**							
Total	1.35	1.06	0.00	7.68	1.13	0.50	1.96
Male	1.50	1.26	0.00	7.68	1.13	0.49	2.21
Female	1.29	0.95	0.01	5.16	1.13	0.51	1.89

AD: average distance, SP: symmetry percentage, CVAS: Chin Volume Asymmetry Score, Pg: pogonion.

**Table 4:** Effect of symmetrical chewing on facial asymmetry using linear regression.

	AD whole face (mm)			AD lower face (mm)		
	Coefficient	CI 95 %	*p*	Coefficient	CI 95 %	*p*
Intercept	0.87	0.79–0.95	**<0.001**	1.32	1.09–1.54	**<0.001**
Symmetrical chewing	0.001	−0.31 to 0.03	0.956	0.02	−0.08 to 0.11	0.727
Sex (female)	−0.13	−0.16 to −0.10	**<0.001**	−0.18	−0.28 to −0.08	**<0.001**
	**SP whole face (%)**			**SP lower face (%)**		
	**Coefficient**	**CI 95 %**	***p***	**Coefficient**	**CI 95 %**	***p***
Intercept	41.33	37.88–44.78	**<0.001**	29.11	22.40–35.82	**<0.001**
Symmetrical chewing	0.76	−0.66 to 2.18	0.295	0.45	−2.31 to 3.21	0.748
Sex (female)	7.32	5.78–8.86	**<0.001**	4.70	1.71–7.69	**0.002**
	**CVAS**			**Pg distance from midline (mm)**		
	**Coefficient**	**CI 95 %**	***p***	**Coefficient**	**CI 95 %**	***p***
Intercept	1.09	1.06–1.12	**<0.001**	1.57	1.17–1.96	**<0.001**
Symmetrical chewing	0.007	−0.01 to 0.02	0.285	0.09	−0.07 to 0.26	0.273
Sex (female)	−0.002	−0.02 to 0.01	0.824	−0.20	−0.38 to −0.02	**0.025**

AD: average distance, SP: symmetry percentage, CVAS: Chin Volume Asymmetry Score, Pg: pogonion, CI: confidence interval.

Self-reported TMD pain has a statistically significant influence on the sidedness of the chin (Table 5). The odds of having a larger side of the chin on the same side as self-reported TMD pain is 9.45 times higher. Other variables in Table 5 do not have a statistically significant impact.

**Table 5:** Estimating variables affecting sidedness of chin by using logistic regression.

Sidedness of chin	Coefficient	Odds ratio	CI 95 %	*p*
Intercept	0.51	1.67	1.09–2.56	**0.020**
Wear	0.04	1.04	0.81–1.34	0.775
PCS	−0.16	0.85	0.68–1.08	0.179
Fillings	−0.07	0.93	0.83–1.04	0.190
Caries	0.02	1.02	0.93–1.13	0.654
Extracted teeth	0.12	1.13	0.75–1.71	0.563
TMD pain, self-reported	2.25	9.45	1.20–74.63	**0.033**

CI: confidence interval, PCS: preferred chewing side, TMD: temporomandibular disorders.

Caries: includes also tooth cracks.

Wear: attrition and erosion.

Odds ratio: > 1 increases the odds of having a bigger side of the chin on the same side as the variable.

## Discussion

According to this study, the variable most affecting PCS is extracted teeth. Subjects prefer chewing on the side with more teeth. The odds of having more missing teeth and PCS on opposite sides is 2.44 times higher when one tooth is missing and even exp(2*0.89) = 5.93 times higher in the case of two missing teeth. This is understandable because masticatory performance is usually better on that side when there are no unnecessary gaps. Missing teeth on the left side increases both sides chewing, and one reason for that could be that maybe chewing is weighted to the right side among both side chewers. Some of the previous studies have found that missing teeth or implants do not have an impact on PCS [26, 35], but others have found that asymmetric tooth loss can lateralise chewing pattern [36]. The present study agrees with that. Dental implant works like real tooth, so its effect on PCS is not that much, obviously. There is disagreement about the influence of wear on PCS, because asymmetric wear has not been found to affect PCS, or PCS and wear have been found on the same side [24, 36]. Caries is not related to PCS according to literature [25, 37]. Present study did not find evidence between PCS and wear, either. Differences between sexes were not found, and there is no consensus about sex and PCS association in the literature either [23, 35].

Sex of the subject has an impact on facial asymmetry: all six asymmetry scores showed that females have more symmetry compared to males in the area of the lower face and the whole face as well. The results are similar to previous studies [2, 19, 38]. Males and females have different soft tissue profiles, which may have some effects. Males tend to have more prominent faces, whereas females have more round faces and the amount of soft tissue is greater compared to males. Soft tissue could reduce the difference in asymmetry of the skeletal structures. Generally, greater facial volume and bigger size of the lower jaw in males compared to females could lead to greater amount of facial asymmetry. Also, facial growth is bigger in males compared to females, which can lead to highlight asymmetrical features.

PCS does not have an influence on contralateral chin side. Instead, an earlier study with twins found the association between PCS and the sidedness of chin [2]. Self-reported TMD pain has an impact on the sidedness of the chin. Model shows that there is more self-reported TMD pain in the larger side of the chin (OR = 9.45). It has been reported that asymmetrical chewers have more signs and symptoms of TMD, and unilateral temporomandibular joint diseases are associated with facial asymmetry [39–41]. The distance from the Menton landmark to facial midline is significantly reduced in patients with disk displacement compared to patients with normal disc position. Our finding is even more sensitive because just a reported pain affects facial asymmetry. Originally, TMD was decided to include as a confounding variable when studying the influence of PCS on facial asymmetry. However, the possible effect that was found gave us ideas for the next study, where we will examine the effect of TMD on facial asymmetry in more detail.

The weakness of this study concerns the different chewing materials. Three different chewing particles were used during the study, which may have affected the results. Anyhow, different chewing materials had no significant impact on the distribution of chewing sides. In any case, all the materials were hard, which gives a better indication of PCS compared to soft food [42]. Subjects have answered TMD pain questions independently, so answers may not be reliable. Further, PCS was only studied at one time point on command alongside other examinations, which may have impact on results. Time of tooth loss was not considered, which can complicate the assessment of the results. It would have required a different research design, but in the future, it will be better considered.

## Supplementary Material

Chewing side preference, facial asymmetry and related factors in the Northern Finland birth cohort 1986
