# Supplementary material for: Chewing side preference, facial asymmetry and related factors in the Northern Finland birth cohort 1986
Source: Acta Odontol Scand. 2024 Sep 18;83:41392. doi: 10.2340/aos.v83.41392 (PMC11423695; doi:10.2340/aos.v83.41392)
Supplement: Chewing side preference, facial asymmetry and related factors in the Northern Finland birth cohort 1986 [file AOS-83-41392-s1.pdf]

Supplementary material has been published as submitted. It has not been copyedited or typeset by Acta Odontologica Scandinavica.

Supplementary Material

Supplementary table 1. Prevalence of TMD diagnoses in Northern Finland Birth Cohort (NFBC) 1986 study.

Diagnosis	Total (n=748)	Male (n=296)	Female (n=452)
**DD with reduction**			
Both sides n (%)	10 (1.3)	0 (0.0)	10 (2.2)
Unilateral n (%)	37 (4.9)	9 (3.0)	28 (6.2)
**DD without reduction with limited opening**			
Both sides n (%)	1 (0.1)	0 (0.0)	1 (0.2)
Unilateral n (%)	2 (0.3)	0 (0.0)	2 (0.4)
**Degenerative joint disease**			
Both sides n (%)	5 (0.7)	2 (0.7)	3 (0.7)
Unilateral n (%)	22 (2.9)	5 (1.7)	17 (3.8)
**Myofascial pain with referral**			
Both sides n (%)	6 (0.8)	1 (0.3)	5 (1.1)
Unilateral n (%)	19 (2.5)	3 (1.0)	16 (3.5)
**Myalgia**			
Both sides n (%)	31 (4.1)	6 (2.0)	25 (5.5)
Unilateral n (%)	48 (6.4)	7 (2.4)	41 (9.1)
**Arthralgia**			
Both sides n (%)	5 (0.7)	2 (0.7)	3 (0.7)
Unilateral n (%)	10 (1.3)	1 (0.3)	9 (2.0)
**Headache attributed to TMD**			
Both sides n (%)	12 (1.6)	3 (1.0)	9 (2.0)
Unilateral n (%)	24 (3.2)	2 (0.7)	22 (4.9)

DD: disc displacement, TMD: temporomandibular disorders

Supplementary table 2. Effect of different variables on PCS using multinominal logistic regression.

PCS	Coefficient	Odds ratio	CI 95 %	p Value
**Right vs left side**				
Intercept	-0.95	0.39	0.17-0.87	**0.021**
Fillings	-0.10	0.90	0.77-1.05	0.194
Caries	0.15	1.16	0.86-1.57	0.330
Wear	0.17	1.19	0.84-1.68	0.325
Extracted teeth	0.89	2.44	1.35-4.40	**0.003**

TMD diagnoses	-0.30	0.74	0.46-1.19	0.214
Sex	-0.18	0.83	0.51-1.35	0.453
**Right vs both sides**				
Intercept	0.28	1.33	0.77-2.29	0.310
Fillings	-0.04	0.96	0.86-1.07	0.432
Caries	-0.00	1.00	0.81-1.23	0.984
Wear	0.08	1.09	0.85-1.38	0.509
Extracted teeth	0.02	1.02	0.69-1.51	0.922
TMD diagnoses	-0.25	0.78	0.56-1.08	0.135
Sex	-0.16	0.86	0.62-1.19	0.351

PCS: preferred chewing side, CI: confidence interval, TMD: temporomandibular disorders
Caries: includes also tooth cracks
Wear: attrition and erosion
Odds ratio: > 1 increases the odds of having PCS on the opposite side of the variable

Supplementary table 3. Effect of different variables on PCS using multinomial logistic regression.

PCS	Coefficient	Odds ratio	CI 95 %	p Value
**Left vs right side**				
Intercept	-0.95	0.39	0.17-0.87	**0.021**
Fillings	-0.10	0.90	0.75-1.05	0.194
Caries	0.15	1.16	0.86-1.57	0.330
Wear	0.17	1.19	0.84-1.68	0.325
Extracted teeth	0.89	2.44	1.35-4.40	**0.003**
TMD diagnoses	-0.30	0.74	0.46-1.19	0.214
Sex	-0.18	0.83	0.51-1.35	0.453
**Left vs both sides**				
Intercept	-1.23	0.29	0.13-0.65	**0.002**
Fillings	-0.06	0.94	0.80-1.10	0.440
Caries	0.15	1.16	0.86-1.57	0.317
Wear	0.09	1.10	0.79-1.53	0.590
Extracted teeth	0.87	2.39	1.33-4.30	**0.004**
TMD diagnoses	-0.05	0.95	0.59-1.53	0.836
Sex	-0.03	0.97	0.60-1.56	0.904

PCS: preferred chewing side, CI: confidence interval, TMD: temporomandibular disorders
Caries: includes also tooth cracks
Wear: attrition and erosion
Odds ratio: > 1 increases the odds of having PCS on the opposite side of the variable
